# Relationship between biometeorological factors and the number of hospitalizations due to asthma

**DOI:** 10.1038/s41598-020-66746-8

**Published:** 2020-06-12

**Authors:** Anna Romaszko-Wojtowicz, Iwona Cymes, Ewa Dragańska, Anna Doboszyńska, Jerzy Romaszko, Katarzyna Glińska-Lewczuk

**Affiliations:** 1 Faculty of Health Sciences, Department of Pulmonology, University of Warmia and Mazury in Olsztyn, Olsztyn, Poland; 2 Department of Water Resources, Climatology and Environmental Management, University of Warmia and Mazury in Olsztyn, Olsztyn, Poland; 3 School of Medicine, Department of Family Medicine and Infectious Diseases, University of Warmia and Mazury in Olsztyn, Olsztyn, Poland

**Keywords:** Asthma, Climate and Earth system modelling

## Abstract

The incidence of asthma exacerbation depends on atmospheric conditions, including such meteorological factors as the ambient temperature, relative air humidity or concentration of atmospheric aerosols. An assessment of relations between the frequency of asthma exacerbation and environmental conditions was made according to the meteorological components, the biometeorological index *UTCI* (Universal Thermal Climate Index), as well as selected air quality parameters, including concentrations of PM_10_ and PM_2.5_. The study was conducted on the basis of a retrospective analysis of medical data collected at the Independent Public Hospital of Tuberculosis and Pulmonary Diseases in Olsztyn (Poland). Our analysis of patient data (from 1 January 2013 until 31 December 2017) showed a significant correlation between the number of asthma exacerbation and the *UTCI* value. More frequent asthma exacerbations are observed in patients aged over 65 years when air humidity increases. The *UTCI* values contained within class 5, describing thermoneutral conditions, correspond to an average frequency of asthma exacerbation. A decline in the *UTCI* value leads to a reduced number of asthma exacerbation, while a rise makes the cases of asthma exacerbations increase.

## Introduction

According to the definition provided by the Global Initiative for Asthma (GINA), asthma is a heterogenous disease induced by an inflammatory condition in the airways^1^. It is characterized by reversible respiratory obstruction, which is accompanied by such symptoms as cough, dyspnea and thoracic pain. The disease most often affects children and elderly people^2^. The mean incidence is 3–5% in developing countries and >20% in developed ones^3^. The available data as of 2016 allows us to estimate that globally around 339.4 million people suffer from asthma^4^. In Poland, the affected people represent circa 5–7% of the adult population, i.e. around 2 million people^5^. With regard to its etiology, asthma is divided into allergic (first symptoms typically occur in childhood) and non-allergic one (usually developing in adulthood). The etiopathogenesis of asthma takes into account the influence of such environmental factors as temperature, atmospheric pressure, air humidity, indicators of air pollution: ozone, carbon dioxide, sulphur dioxide and particulate matter^6–10^. Atmospheric aerosols, PM_2.5_ and PM_10_, are a mixture of air suspended particles with a diameter of no more than 2.5 and 10 µm, respectively. In susceptible people, particulate matter is responsible for respiratory tract symptoms such as cough and wheezing^11,12^. In vulnerable patients, they give rise to respiratory tract symptoms, including cough and wheezing.

The human organism is exposed to the influence of various elements of the atmospheric environment, which shape the thermal comfort of one’s body, affect well-being, and even influence health. A variety of methods and indices are employed in biometeorology to determine the relationship between atmospheric factors and human health^13^. An indicator which has been developed to achieve a standard measure for the assessment of external environmental conditions in various spatial scales is the *UTCI* (Universal Thermal Climate Index)^14,15^. This index is based on an analysis of the thermal balance of a human body, made according to the Fiala’s multi-node model of human heat transfer, providing information on actual body temperature regulatory processes, which are dependent on the ambient meteorological conditions^16–18^. In comparison with other biometeorological parameters, the *UTCI* is more sensitive to even small changes in temperature, solar radiation, air humidity and wind velocity^15^.

Changes in climate have always had and will continue to have strong influence (direct and indirect) on many sectors of economy and on other areas of human activity. Their impact will become stronger as the incidence of extreme weather events increases, and whenever there are situations where the weather typical of a given area occurs in an untypical season of the year (for example very warm winters in Poland). Research is undertaken to explore how the climate changes observed may affect the health of human populations in particular countries as well as globally^19–23^.

The intricate influence of climatic changes on the epidemiology of respiratory tract disorders has not been completely identified. The effect of global warming on the onset, duration and intensity of pollen season, associated with the incidence and severity of asthma exacerbation, has been described as well as the relationship with air pollution and infections of the airways^24,25^. Both short- and long-term exposure to PM_2.5_ and PM_10_ can be linked to a more frequent incidence of asthma symptoms or less effective asthma control^26,27^.

It was evidenced in the 1990s that both high and low air humidity, elevated atmospheric pressure, low ambient temperature, rainfalls and storms all had an effect on the occurrence of dysfunctions of the respiratory system^28–31^. Analysis of data obtained from literature references pertaining to the effect of ambient temperature on the occurrence of acute asthma exacerbation provides us with contradictory information. Fitzgerald *et al*. reports that high temperature contributes to more frequent asthma exacerbations, while Soneja *et al*. implicates such impact of low temperature^32,33^. Kaminsky *et al*. demonstrate that a change in ambient temperature may cause the activation of inflammatory pathways, inducing hyperactivity of bronchi, their remodeling and the narrowing of the airways, which consequently stimulates asthma exacerbation^34^. In turn, Epton *et al*. maintain that a rise in temperature can also contribute to a higher incidence of asthma exacerbations due to greater exposure to allergens^35^.

Ehara *et al*. showed a positive relationship between the number of patients admitted to hospital with exacerbated asthma and a rise in atmospheric pressure as well as a decrease in the relative air humidity^36^. In another paper, Abdullah M. Al-Rubaish described the effect of wind blasts during a storm carrying bioaerosol, which due to a sudden release of spores and pollen contributed to the occurrence of asthma symptoms^37^.

To the best of our knowledge, no attempt has been made so far to describe an application of any complex biometeorological index to an analysis of factors affecting the incidence of asthma exacerbations. In our article, we suggest using the *UTCI*, an index measuring human thermal comfort as a complex and universal biometeorological indicator in an assessment of the frequency of asthma exacerbation. Another objective of this research was to analyze the incidence of asthma exacerbations relative to such atmospheric environmental factors as: temperature and humidity of air, wind velocity, and particulate matter parameters PM_2.5_ and PM_10_.

## Material and Methods

### Study population

The study was conducted on the basis of a retrospective analysis of medical data collected at the Independent Public Hospital of Tuberculosis and Pulmonary Diseases in Olsztyn (Poland) between 1 January 2013 and 31 December 2017. The hospital is the leading reference centre for pulmonary diseases in the Province of Warmia and Mazury. The patients included in the study were selected according to the ICD10 classification (comprising codes from group J45 or coexisting with J45 diseases). A database was created, containing 1 449 records (14.12% of the total number of 29 088 patients hospitalized in this period), including 574 (39.6%) records of patients aged over 65 years. Each case of hospitalization when asthma was not determined as the primary cause for hospitalization was verified based on the patient’s medical record for its exacerbation. The mean age of the entire population was 61.9 (±14.3 SD) years, women 61.7 (n = 770; ±14.6 SD) years, and men 62.2 (n = 679;±13.9 SD) years.

### Meteorological data

The meteorological data from the time period subjected to the analysis (2013–3017) were acquired from a meteorological station of the Institute of Meteorology and Water Management in Olsztyn (Poland). These data included values of the mean daily, maximum and minimum temperatures, wind velocity, relative air humidity and atmospheric pressure for each day. The data of 12 UTC (coordinated universal time) regarding the meteorological parameters (air temperature, relative air humidity, actual vapor pressure, cloudiness, wind velocity) served to calculate values of the *UTCI*, whose procedures of calculation were provided by Fiala and Brode^18,38^. The *UTCI* is based on objective changes in an organism’s physiological parameters in response to environmental conditions. Values of the *UTCI* are expressed in degrees Celsius [°C], and are a measure of thermal load onto the human body.

On days with low air temperature, at high wind velocity, cold stress occurs (cold stress classes from 1 to 5 – the *UTCI* values ≤9.0 °C), whereas days with high air temperature and intensive solar radiation are conducive to heat stress (heat stress classes from 7 to 10, values of the *UTCI* > 26 °C). Thermal comfort conditions (thermoneutral) are ones where values of the *UTCI* are in a range of 9.1 a 26 °C (class 6)^38^.

The area covered by the research, according to the updated Köppen-Geiger climate classification, lies in the sphere of continental climates, without dry season and with warm summer (Dfb)^39^. It is characterized by high changeability of weather conditions between years, and by distinct seasonability in the course of a year, which determines the presence of 4 seasons of the year. The mean annual air temperature during the period of the study was 8.5 °C, with January being the coldest month having the mean temperature of −3.1 °C, while July was the warmest month, with the mean air temperature of 18.5 °C. The annual precipitation total was 660 mm, and the mean relative air humidity equaled 78%. The mean annual wind velocity reached 3 m/s, with the highest values observed in December and January.

During the analyzed period (01.01.2013–31.12.2017), there were no events of extreme cold stress or extreme heat stress, i.e. class 1 or class 10, respectively. Cold stress conditions occurred during 975 days (53.4%) (Table 1). Thermoneutral conditions appeared in 38.77% of time (n = 708 days), whereas heat stress was in 7.83% (n = 143) of the total days (Table 1). The analysis therefore excluded the *UTCI* classes absent during the research period, i.e. classes 1 and 10 (extreme cold stress and extreme heat stress), nor did it take into account classes 2, 8, 9 (very strong cold stress, strong heat stress, very strong heat stress, respectively), which did not coincide with any case of asthma exacerbation. Exacerbations of asthma appeared in patients on days corresponding to classes 3–7 of the *UTCI*.

**Table 1:** Characteristics and frequency of UTCI classes in the study period (2013–2017).

Thermal conditions	Cold stress					Thermo-neutral conditions	Heat stress			
Class name	extreme	Very strong	Strong	Moderate	Slight		Moderate	Strong	Very strong	extreme
Range of *UTCI* values (°C)	<= −40.0	−39.9 to −27.0	−26.9 to −13.0	−12.9 to 0.0	0.1 to 9.0	**9.1 to 26.0**	26.1 to 32.0	32.1 to 38.0	38.1 to 46.0	>46.0
Class of thermal stress	**1**	**2**	**3**	**4**	**5**	**6**	**7**	**8**	**9**	**10**
Number of days	0	3	62	482	428	708	108	33	2	0
Percentage of the total	0	0.16	3.40	26.40	23.44	38.77	5.91	1.81	0.11	0

The World Health Organization recommends a very strict approach to the emission of particulate matter, where the allowable daily concentration for PM_10_ is 50 µg/m^3^ and for PM_2.5_ 25 µg/m^3^ ^40^. During the analyzed time period, the average annual number of days when the permissible concentrations of PM_2.5_ and PM_10_ were exceeded equaled 2 and 6 days, respectively.

The exceeded threshold of PM_10_ and PM_2.5_ concentrations appeared only during the house heating season, i.e. from late autumn to early spring (October–March), with the highest incidence in the months from December to March, when it happened on an average of 3 and 11 days, respectively for PM_10_ and PM_2.5_.

### Statistical analysis

Statistical analysis was performed to reveal the relationship between asthma exacerbation cases among the hospitalized patients and meteorological variables as well as air pollutants. The explanatory variables (daily mean air temperature, *UTCI* and *UTCI* classes 3–7, wind velocity, relative air humidity, atmospheric pressure, rainfall sum, PM_2.5_ and PM_10_) were employed for the Generalized Linear Model (GLM) assuming the Poisson’s distribution and using a logarithm as the link function^41^. The contribution of the predictors for two groups of patients with asthma, aged <= 65 and >65, was evaluated by testing individual coefficients with the Wald’s test at p ≤ 0.05.

For the data observed to be considered statistically significant, the ±95% confidence interval (CI) was calculated. Statistical analyses were performed with STATISTICA 13.1 for Windows.

### Ethics statement

The study presented in this paper is a retrospective analysis of data from medical histories of patients. The data required to make the analysis were collected having received the consent of the hospital’s authorities. The researchers did not use or process any personal data of the hospitalized patients. The research did not require a permit from a bioethics committee.

All the experimental protocols for involving human data in the study were in accordance with national Polish guidelines. According to Ethics Committee of the University of Warmia and Mazury in Olsztyn, Poland this research, as a blinded retrospective analysis, does not required a separate consent from the bioethics committee. Informed consent for subject was waived by the Ethics Committee of the University of Warmia and Mazury in Olsztyn, Poland.

### Exemption for the study

According to Ethics committee University of Warmia and Mazury In Olsztyn, Poland this research does not meet the criteria of a medical experiment and therefore does not require a separate consent from the bioethics committee.

### Consent waiver

According to Ethics committee University of Warmia and Mazury In Olsztyn, Poland this research, as a blinded retrospective analysis, does not required a separate consent from the bioethics committee.

## Results

Table 2 presents the analysis of covariance between the number of hospital admissions due to asthma (exacerbations and *de novo* diagnosis) and the analyzed biometeorological factors. From among categorical predictors (UTCI classes), class 6 representing thermoneutral conditions was considered as the baseline level. To recognize the effect of other meteorological parameters and air pollution on the quality of the model (GLM) we employed the following continuous covariables: average wind velocity, average air humidity, average daily temperature, average atmospheric pressure, daily rainfall total, average PM_2.5_ concentration and average PM_10_ concentration (atmospheric aerosols containing particles with a diameter of no more than 2.5 µm and 10 µm, respectively).

**Table 2:** Results of ANCOVA for a generalized linear Poisson log model showing the relationship between the number of asthma hospital admissions and ambient meteorological parameters as well as air pollutants.

Variable	Estimate	Wald’s test	*p*-value	Estimate	Wald’s test	*p*-value	Estimate	Wald’stest	*p*-value
	Total			Age ≤65			Age > 65		
Intercept	3.23	0.50	0.477	1.21	0.04	0.840	5.23	0.57	0.452
*UTCI* Class 3	**−0.81**	**7.36**	**0.007**	**−**0.72	3.41	0.065	**−0.92**	**3.83**	**0.049**
*UTCI* Class 4	**−**0.33	3.37	0.067	**−**0.31	1.84	0.175	**−**0.35	1.51	0.219
*UTCI* Class 5	**−**0.14	1.35	0.245	**−**0.15	0.92	0.339	**−**0.13	0.47	0.494
*UTCI* Class 7	0.23	2.81	0.093	**0.40**	**5.97**	**0.015**	**−**0.14	0.28	0.596
*UTCI* Class 8	**−**0.04	0.00	0.950	0.14	0.04	0.845	**−**0.27	0.07	0.791
Air temperature	**0.17**	**16.86**	**0.000**	**0.10**	**3.97**	**0.046**	**0.26**	**16.19**	**0.000**
Relative humidity	**−0.03**	**24.35**	**0.000**	**−**0.01	3.41	0.065	**−0.04**	**29.60**	**0.000**
Rainfall sum	0.00	0.48	0.487	0.00	0.76	0.382	0.00	0.06	0.803
Wind speed	**−**0.06	1.25	0.263	**−**0.07	0.91	0.341	**−**0.06	0.44	0.507
Atmospheric pressure	0.00	0.11	0.736	0.00	0.11	0.740	0.00	0.00	0.984
PM_2.5_	0.01	1.86	0.173	0.02	3.70	0.054	0.00	0.03	0.868
PM_10_	**−**0.01	1.16	0.281	**−0.02**	**6.59**	**0.010**	0.01	1.53	0.216

Thermoneutral conditions (UTCI class 6) were set up as the baseline level. Significant values at p ≤ 0.05 are depicted in bold, Cl ± 95%.

The analysis of the frequency of hospital admissions due to asthma, against the background of meteorological parameters and mutual interactions between these variables, shows that it is reversely proportional to the *UTCI* values and average relative humidity of air (p = 0.030 and p = 0.002, respectively).

**Figure 1:**
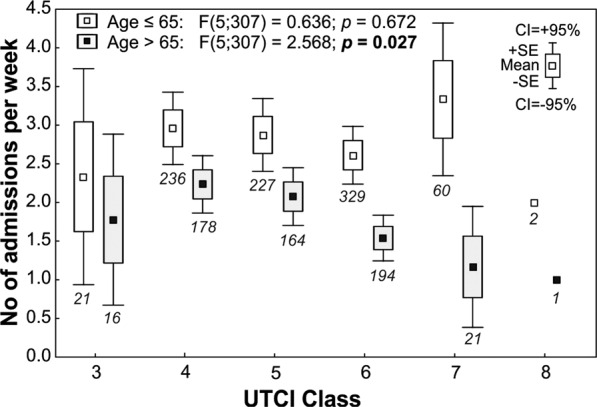
Frequencies of hospital admissions due to asthma in age groups ≤65 and >65 years in relation to UTCI classes in the period of observation. Numbers below whiskers denote the number of patients hospitalized.

Moreover, it has been demonstrated that the distribution of incidence of asthma exacerbation is related to the *UTCI* classes 3 and 7. The distribution of data in ranges of confidence intervals (CI = ±95%) is illustrated in Fig. 1.

**Figure 2:**
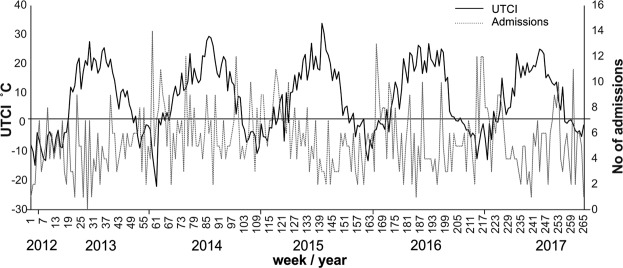
Actual number of patients presenting with exacerbated asthma versus the course of UTCI values during the analyzed time period.

Figure 2 shows the distribution of asthma exacerbation against the backdrop of the course of mean daily *UTCI* values in the multi-annual period of 2013–2017. The sinusoidal course of both curves depicts a reverse amplitude of the course of the *UTCI* coefficient and the occurrence of asthma exacerbations. In the summer period, extremely high *UTCI* values, i.e. heat stress, are accompanied by ‘peaks’ in the incidence of asthma. In winter, due to cold stress, there is an increase in the number of asthma exacerbations.

Furthermore, the analyzed population was divided into two age groups: aged ≤65 years and >65 years of life (Table 2). The incidence of exacerbated asthma among younger patients was also higher at higher *UTCI* coefficients (p = 0.015), that is when heat stress occurred.

**Figure 3:**
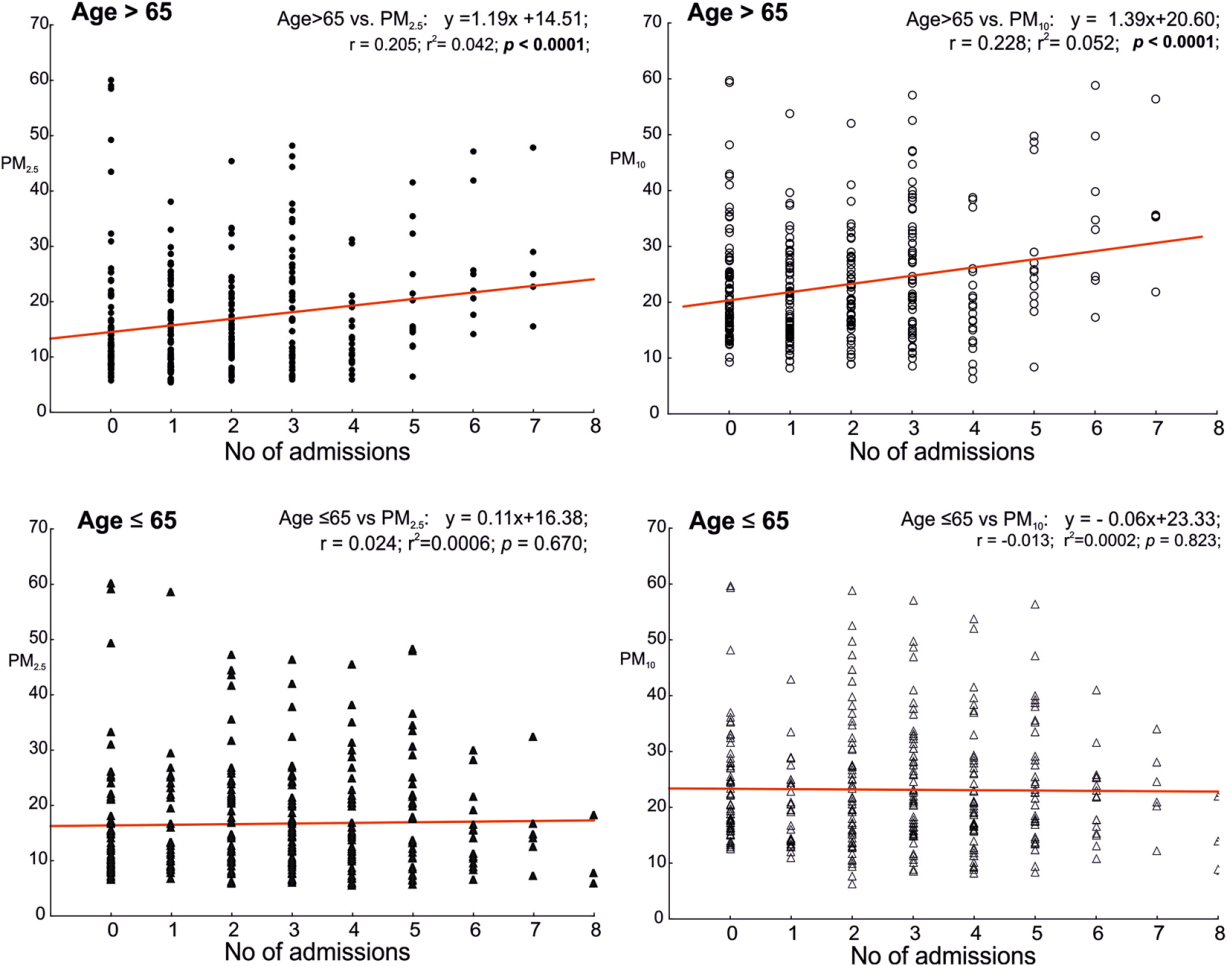
Numbers of hospital admissions due to asthma for two age groups (≤65 and >65) in relation to PM2.5 and P10 concentrations in the air.

In turn, the subpopulation of patients aged over 65 years was found to present a statistically significant negative relationship between the frequency of asthma exacerbation and the presence of strong cold stress. Regression analysis revealed a direct relationship between the number of hospitalizations due to asthma and concentrations of PM_2.5_ and PM_10_; however, only in the older patient group (Fig. 3).

**Figure 4:**
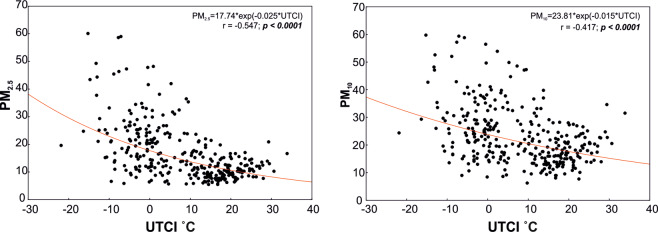
Regressions curves between UTCI and concentrations of PM2.5 (left graph) and P10 (right graph) in the air.

Analogous regression analysis performed for relative humidity did not reveal statistically significant correlations (not shown in the graph). It should be stressed that concentrations of PM_2.5_ and PM_10_ are higher with lower outdoor temperature and lower *UTCI* (Fig. 4).

## Discussion

Research dealing with the influence of meteorological factors on the incidence of exacerbation of asthma and other respiratory tract diseases is gaining greater interest ^42,43^. Most researchers approach the issue through the prism of atmospheric contamination. Papers referring to basic or more complex biometeorological parameters are less numerous, and we were unable to detect a single study that would employ the *UTCI* for this purpose.

It should be stressed that the impact of global warming on local atmospheric conditions, and as a further consequence on the epidemiology of various diseases, depends on the geographical location of a study, that is on the local climate.

Considering the baseline level of UTCI class 6 as thermoneutral conditions (ANCOVA, p < 0.05), statistically higher *UTCI* values (class 7) and an increase in the mean daily temperature (Table 2) predispose to a higher number of hospital admissions (age ≤ 65) due to asthma. On days identified as belonging to *UTCI* class 3, that is on days with strong cold stress, the incidence of asthma exacerbation declines particularly among the elderly (Table 2). In our opinion, this dependence is direct and it is not distorted by changes in atmospheric dust concentrations. Although the concentrations of PM_10_ and PM_2.5_ found in Poland are among the highest in the European Union countries, events of excessive particulate matter concentrations in air in Olsztyn (a beneficial geographical location, favoring air circulation) were noted only sporadically^44^. This enabled us to reveal in our data the direct impact of cold stress and heat stress on the function of the respiratory system. The results of ANCOVA analysis revealed that the impact on the *UTCI* class on the number of hospitalizations due to asthma may be limited by the increase in the concentration of PM_10_ (covariate variable) in the air (Table 2). Higher concentrations of PM_2.5_ and PM_10_ occuring in lower air temperatures, and thus at the lower *UTCI* (Fig. 4) may increase the number of hospitalizations due to asthma (Fig. 3), especially among older patients. This effect is, however, insufficient to eliminate the impact of cold stress.

In a number of articles dedicated to cold-induced asthma, authors focus on the influence of cold air on airway patency^45^. The spastic effect induced by cold air is particularly well known among athletes, when during physical exertion, as a result of hyperventilation, and through an increase in the osmolarity of fluid in airways, the direct influence of cold air on reducing the patency of bronchi becomes distinctly manifested^46^. When cold stress is present, the breathing pattern is altered. Diesel *et al*. demonstrated that a decrease in ambient temperature from 18 to 4 °C led to a decrease in respiratory rate by 25%, while tidal volume rose by 35%^47^. An average person exposed to cold stress tries to breath more slowly and deeply rather than hyperventilating. Moreover, a change in air temperature, under physiological conditions with no physical effort, is set off by the upper airways (the nose) and has no effect on the lower respiratory tract. Sthurman-Ellstein *et al*. showed that if asthma patients were breathing through the nose during an effort stress test, there was no bronchial constriction appearing in response to the physical effort^48^. Bronchoconstriction is unobserved in people taking ice baths either^49^. Moreover, an increase in the level of catecholamines, which are expected to widen the bronchi, is emphasized. The *UTCI*, an indicator developed to describe the impact of atmospheric conditions on the human body, reflects very well the influence of both cold and heat stress. Basic parameters, such as ambient temperature, wind velocity, relative air humidity, are sufficient to describe the weather conditions, but the *UTCI* proves to be a valuable tool in the type of research presented in this article. To the best of our knowledge, this is the first study that describes the relationship between the cases of asthma exacerbations and the *UTCI*.

In their meta-analysis of 81 studies, Cong *et al*. noted that a decrease in temperature contributed to a higher incidence of asthma cases, but only in a population of children, while no such dependence was observed among adults^50^. This finding among the under-age population might be associated with greater physical activity among children, which more often causes hyperventilation (same as among athletes), especially when we consider the fact that air flow through children’s respiratory tract takes a shorter time (the anatomical structure).

These epidemiological data, not entirely coherent, might have other roots in the epidemiology of upper respiratory tract infections. Infections with Rhinovirus (RV) are responsible for over half of cases of URT infections, and may cause episodes of asthma in predisposed patients^51^. Such infections occur in mass numbers in our climatic zone after the first autumn weather break. However, their further spread does not depend on the weather conditions, but progresses in line with the general laws of epidemics^52^.

Global urbanization also leads to an increase in the environmental pollution caused by combustion of fossil fuels, biomass, emissions from agriculture, and windborne mineral dusts as well as organic matter. Solid particles in air, such as PM_2.5–10_, that is a mixture of particles emitted from numerous sources, may contribute to the activation of cytokine pathways and activation of an inflammatory response in the airways^53,54^. PM_2.5_ alone can induce an allergic reaction due to overproduction of IgE mediated by Th2 and Th17 lymphocytes, and thus contribute to a higher incidence of asthma exacerbations^55,56^. Based on the data submitted to our analysis, we have been able to demonstrate that a higher PM_10_ concentration leads to an increased frequency of acute asthma exacerbation in elderly patients (Table 2). With age, the epithelium of bronchi becomes thinner, which means that particles larger in diameter can penetrate with air into the bronchi, settle down in the airways and induce local inflammatory response^57,58^.

Global warming entails longer pollen seasons, increased amounts of pollen produced and its higher allergenicity^59^. These factors add to a rise in the incidence of asthma exacerbation^59,60^. Higher air temperatures in winter, earlier spring season, earlier autumn, as well as delayed winter lengthen the duration of exposure to fungal endospores. Particulate matter and mould spores are basic allergens, implicated as factors in the etiopathogenesis of asthma^8^. Particulate matter, that is fine powder-like substances with grains of the size 2–10 µm in diameter (PM_2,5_; PM_10_) can easily permeate into the airways. They are also easily carried by wind over large distances and therefore can affect people being far from their source of emission. One of the findings reported by Ziska L *et al*., who in 2009 analyzed data from several centres (8 located in the USA and 2 from Canada), there was the increased length of ragweed pollen season by over 3 weeks in comparison with the year 1995^59^. In Poland, it has been proven that the season of pollination by autumn weeds has been prolonged in the past few decades, mainly as a result of the winter-autumn period lasting longer^61^.

Literature provides contradictory information on the relationship between asthma morbidity and air humidity. Some researchers notice a significant increase in asthma exacerbation cases under the weather conditions characterized by higher ambient air humidity, and link this fact to higher counts of fungal spores^61–63^. Kwon *et al*. associated this phenomenon with the occurrence of fog, which often accompanies higher air humidity, and which alone – by being acid aerosol – is yet another air pollutant^64^. Others emphasize an increase in hospital admissions due to asthma on days with lower air humidity^65^. Our regression analysis performed for air pollution humidity and the number of asthma exacerbations (Fig. 3) does not indicate a direct statistically significant (p < 0.05) relationship between these variables. However, relative humidity (as a covariate) weakens (p < 0.001) the correlation between the *UTCI* class (fixed effect) and the number of asthma cases, as was revealed by covariance analysis (Table 2).

## Conclusions

The increase in the number of hospitalizations due to asthma is related to *UTCI* values. In moderate heat stress (*UTCI* class 7) the frequency of asthma cases increases in younger patients. The number of hospital admissions decreases with a decrease of the *UTCI* value to class 3, that is in the conditions of strong cold stress, especially among patients over 65 year old. This happens despite increased concentrations of PM_2.5_ and PM_10_ in wintertime, that are directly responsible for the increase in hospitalizations due to asthma among these patients.

## Limitations

The results presented above originate from the data collected from one centre, the Independent Public Hospital of Tuberculosis and Pulmonary Diseases in Olsztyn. Characteristic symptoms of asthma such as dyspnea and chest tightness require a differential diagnosis for cardiological and pulmonary causes. Due to the inadequate health care system, patients with these symptoms are first admitted to Emergency Departments in different hospitals, where they are diagnosed and then referred to Pulmonary Departments. This can translate into 1- to 2-day delays in our data. Hence, all meteorological  parameters included in our study were analyzed in weekly intervals rather than on a daily basis. It was important for us in this study to focus attention on an older age group of patients; hence we do not present analyses as regards other possible age subgroups, analyses regarding gender etc.

## Data Availability

The data that support the findings of this study are available on request from the corresponding author.
